# The Effects of Bromocriptine on Preventing Postpartum Flare in Systemic Lupus Erythematosus Patients from South China

**DOI:** 10.1155/2015/316965

**Published:** 2015-04-20

**Authors:** Qiu Qian, Liang Liuqin, Li Hao, Yuan Shiwen, Zhan Zhongping, Chen Dongying, Lian Fan, Xu Hanshi, Yang Xiuyan, Ye Yujin

**Affiliations:** Department of Rheumatology & Clinical Immunology, The First Affiliated Hospital, Sun Yat-Sen University, Guangzhou 510080, China

## Abstract

*Objective*. Prolactin plays an important role on the disease flare of postpartum SLE patients. 76 pregnant SLE patients were enrolled in this study to evaluate the efficacy of bromocriptine (an inhibitor of prolactin secretion) on preventing the postpartum disease relapse.* Methods*. Patients were randomly divided into the treatment group (bromocriptine, 2.5 mg oral, twice a day for 14 days after delivery) and the control group. All the patients were followed up for 12 months. Clinical features were recorded every 4 weeks. Serum prolactin and estradiol levels were measured at the second week and the second month after delivery. The endpoint of the study was disease relapse and defined when SLEDAI score increased by ≥3 points from the antenatal baseline.* Results*. (1) Serum levels of prolactin and estradiol decreased significantly in bromocriptine treatment group at the second week (*P* < 0.001) and second month (*P* < 0.05) after delivery compared to control group. (2) The relapse rate of the treatment group was lower than the control group (*χ* ^2^ = 4.68, *P* = 0.0305).* Conclusions*. Two weeks of oral bromocriptine treatment in postpartum SLE patients may relieve the disease from hyperprolactinemia and hyperestrogenemia and may be beneficial in preventing the patients from disease relapse.

## 1. Introduction

Many lupus patients experience disease flare in late trimester of pregnancy or puerperium [1]. Recently, a retrospective study of pregnancies in Chinese women with systemic lupus erythematosus (SLE) demonstrated that 26 of 68 pregnancies (38%) who were stable at conception encountered disease flare during pregnancy or postpartum [2]. Therefore, disease flare is a major concern of rheumatologists when facing pregnancy and puerperium SLE patients. High serum levels of sex hormones, especially prolactin and estradiol, have been considered to be associated with the lupus disease activity [3, 4]. Serum levels of prolactin and estradiol are usually increased significantly during pregnancy, puerperium, or postpartum lactation period compared to normal women [5], which indicated that high serum levels of prolactin and estradiol might contribute to lupus flares [4, 6].

Prolactin is a polypeptide hormone secreted by the anterior pituitary gland. It not only regulates the growth and differentiation of the mammary gland and ovary, but also initiates and maintains lactation. Prolactin is considered as well as a cytokine because it is secreted by immune cell and its receptor belongs to the cytokine receptors type 1 family [7]. PRL could enhance the production of autoantibodies by influencing B-cell maturation in the early stage and promoting the survival of self-reactive B cell clones [8]. Bromocriptine, an inhibitor of PRL secretion, is an ergot derivative that binds to dopamine (D2) receptors on normal or adenomatous lactotroph cells. Previous studies demonstrated that bromocriptine could improve the SLEDAI scores in SLE patients with mild to moderate disease activities [9]. The same results were also found in mice [10, 11]. But for postpartum SLE patients, there were few researches on the relationship between prolactin and lupus activity. Our previous work indicated that breast-feeding after delivery had the potential of increasing the incidence of postpartum relapses in disease, and bromocriptine may eliminate this risk [12], but the sample size is small. So in the current study we designed a randomized controlled trial in order to get more information about the efficacy of oral bromocriptine on preventing the postpartum patients with SLE from disease relapse.

## 2. Patients and Methods

### 2.1. Patients

**Figure 1:**
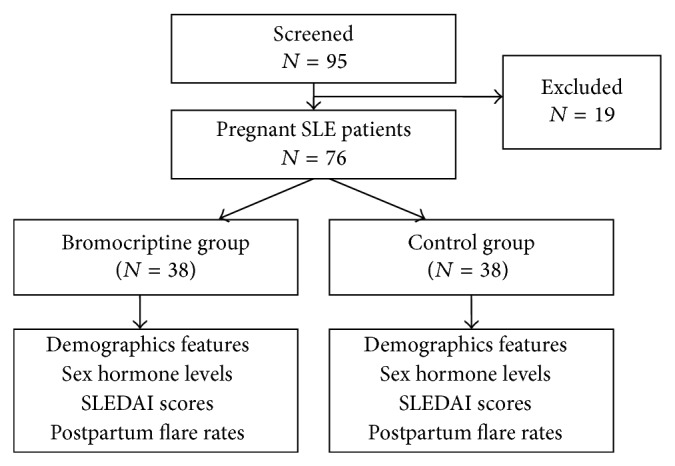
Study design. SLEDAI: systemic lupus erythematosus disease activity index.

95 pregnant SLE patients hospitalized in our hospital between July 2003 and October 2013 were recruited. The study design is presented in Figure 1. Local Ethics Committee approved the protocol and written informed consents were obtained. All patients fulfilled at least 4 of the American College of Rheumatology criteria for SLE [13]. Pregnant lupus patients with inactive or mild-active SLE receiving no more than 10 mg of prednisone (or equivalent) per day were eligible for this study. Disease activity was measured by Systemic Lupus Erythematosus Disease Activity Index (SLEDAI) [14]. Patients were defined as inactive SLE when the SLEDAI scores ≤4 and mild-active with the scores between 5 to 9. Patients were excluded if they (1) had a SLEDAI score ≥10; (2) had proteinuria >500 mg/24 hours or other major organ system involvements; (3) were receiving >10 mg/day of prednisone (or equivalent) during pregnancy; or (4) had a serum level of complement 3 < 0.5 g/L.

### 2.2. Methods

Patients were randomly divided into the bromocriptine group and the control group. The bromocriptine group received 14 days of oral bromocriptine 2.5 mg twice a day within 12 hours after delivery and did not breastfeed their infants. The control group was not treated by bromocriptine or any other medicine that might influence patients' serum prolactin levels.

Demographic data and clinical characteristics were collected 1 to 4 weeks before expected date of childbirth. All patients were followed up every 4 weeks for 12 months. Clinical manifestations and laboratory findings were recorded during every visit to assess the SLE activity. Laboratory investigations included serum levels of antinuclear antibodies, anti-dsDNA antibody, complements, complete blood count, urinalysis, serum albumin, liver function, and creatinine. Systemic Lupus Erythematosus Disease Activity Index (SLEDAI) was used to assess the disease activity. SLEDAI was performed two to four weeks before delivery and every six weeks after delivery. To minimize the influence of observation bias on outcome determination, the SLEDAI scores were calculated by two different investigators. One performed the history collection and physical examination and did not know the serologic findings, and the other one performed the serological assessment and the SLEDAI scoring.

The serum prolactin and estradiol levels were measured twice by radioimmunoassay, at the second week and the second month after delivery, respectively.

### 2.3. End Points of Trial and Definition of Clinical Flares

Increasing of SLEDAI score ≥3 points from antenatal baseline data was defined as the endpoint of this trial—clinical flare. Mild-to-moderate flare was defined if one or more of the following 5 features were fulfilled: (1) an increase of SLEDAI score ≥3 points but <12 points; (2) new/worse discoid, photosensitive, cutaneous vasculitis, bullous lupus, nasopharyngeal ulcers, profundus, pleuritis, pericarditis, arthritis, and fever; (3) an increase in the prednisone dosage but <0.5 mg/kg of body weight per day; (4) initiation of therapy with either hydroxychloroquine or nonsteroidal anti-inflammatory drugs; (5) increase in the physician's global assessment score but no more than 2.5. Severe flares were defined when one or more of the following 4 features were fulfilled: (1) increasing the SLEDAI score ≥12 points; (2) new or worsening central nervous system (CNS) involvement, vasculitis, glomerulonephritis, myositis, thrombocytopenia (platelet count <60 ∗ 10^9^ cells/liter), or hemolytic anemia (Hb < 70 g/L or decreasing of Hb > 30 g/L), required doubling dose of corticosteroid or a final dosage of >0.5 mg/kg/day or hospitalization; (3) any manifestation requiring an increase in the dosage of prednisone or equivalent drug to >0.5 mg/kg/day or initiation of therapy with cyclophosphamide, azathioprine, mycophenolate mofetil, or methotrexate; (4) hospitalization because of lupus activity; or (5) increase in the physician's global assessment score >2.5 [15].

### 2.4. Statistical Analyses

Survival analysis was used to compare disease aggravation and relapse rate between two groups. Log-rank test was used to assess significant differences between two groups. Parameter data, such as SLEDAI score and sex hormone levels, were recorded as X ± SD and tested by *t* test. Nonparameter data were analyzed by Wilcoxon rank sum test. All statistical analyses were performed on STATA 10.0 statistical software.

## 3. Results

### 3.1. The Baseline Demographic Features and Clinical Characteristics of the Patients

Ninety-five pregnant lupus patients were screened, 76 of them qualified for the study and were enrolled into the trial. The patients were randomly divided into the treatment group (*n* = 38) and the control group (*n* = 38). All 38 patients in the treatment group were prescribed with oral bromocriptine for two weeks. There was no statistical difference on the baseline data (demographic features, laboratory findings, and SLEDAI scores) between these two groups (shown in Table 1).

### 3.2. Clinical Outcomes in the Treatment Group and the Control Group

**Figure 2:**
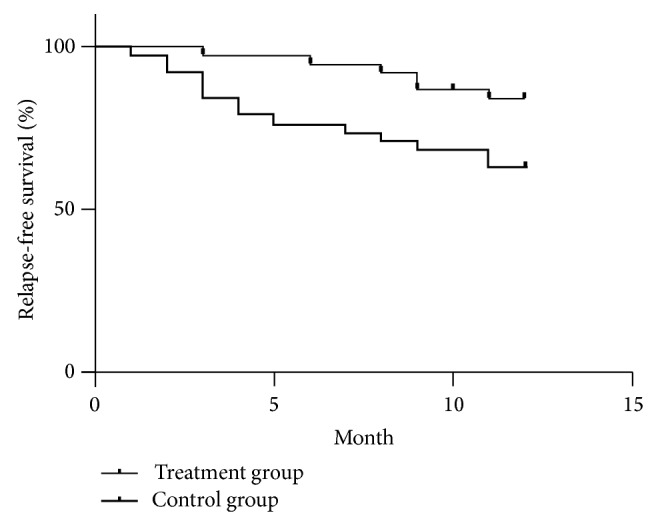
Kaplan-Meier survival curves for relapse-free Survival between the treatment group and the control group.

Twenty pregnant patients experienced 20 flares within 12-month follow-up after delivery. Fifteen cases were mild/moderate flares and five were severe flares. Analysis of flares of any type (mild/moderate or severe) by Kaplan-Meier survival curves found that 6 patients (15.7%) in bromocriptine group and 14 cases (36.8%) in control group experienced at least 1 flare. Log-rank test indicated that there was significant difference on the flare rate between the treatment group and control group (*χ* ^2^ = 4.68, *P* = 0.0305) (shown in Figure 2). Relative risk reduction (RRR) was 68.8% and the 95% confidence interval (CI) was 24.3%–87.1%. Absolute risk reduction (ARR) was 32.4% and the 95%CI was 11.8%–53%. The number needed to treat (NTT) was 3.1, 95%CI (1.9–8.5), which means 3.1 patients needed to be treated to protect one patient from relapse. All severe flares occurred in the control group.

### 3.3. Effect of Bromocriptine on Serum Levels of Prolactin and Estradiol

The serum prolactin and estradiol levels in the treatment group were significantly lower than the levels in the control group at the second week and the second month after delivery, respectively (shown in Table 2). At the end of second week, serum prolactin levels of patients in the treatment group were all in normal range, while those in the control group were higher than the upper limit of normal range (0 versus 100%). At the end of second month, 2 patients in treatment group had increased serum levels of prolactin (5.3%, 2/38), while, in control group, 17 patients experienced hyperprolactinemia (44.7%, 17/38).

### 3.4. Effect of Bromocriptine on Disease Activity of the Patients after Delivery

At the 6th and 12th months after delivery, SLEDAI scores of the treatment group were significantly lower than those of the control group (Table 3).

### 3.5. Cumulative Doses of Immunosuppressive Agents

The cumulative doses of corticosteroid and other immunosuppressive agents after 12-month follow-up were shown in Table 4. There were significant differences on the accumulative doses of prednisone and cyclophosphamide between two groups. In bromocriptine group, the need of immunosuppressants to keep disease-stable was significantly lower than control.

### 3.6. The Adverse Effects of Treatment

Three patients had mild vertigo and nausea in the treatment group and all patients could complete the 2 weeks oral bromocriptine therapy. No severe adverse event was found in this study.

## 4. Discussion

For pregnant SLE patients, though many of them can pull through pregnancy and childbirth in the remission stage of disease, some patients still experience relapse or disease flare during the late trimester of pregnancy or puerperium period. Several studies had shown that hyperprolactinemia was associated with lupus activity in pregnancy. Prolactin can stimulate mammary growth and differentiation, and it progressively increases during these periods [4]. Recently, a study confirmed that prolactin levels are associated with lupus activity, lupus anticoagulant, and poor outcome in pregnancy. Significant linear correlations between prolactin, modified-systemic lupus activity measurement (m-SLAM), and lupus anticoagulant were observed [16]. But for postpartum SLE patients, there were few researches on the relationship between prolactin and lupus activity.

The results in our study showed that the serum prolactin levels in control group were raised obviously at the second week after delivery, about 5 times the upper normal limit of prepregnancy levels. At the second month after delivery, the serum prolactin levels in the control group dropped but still much higher than those in the treatment group. In the control group, SLEDAI scores at the 6th and 12th months after delivery were much higher than those in treatment group. The results of the current study preliminarily indicated that oral bromocriptine for 2 weeks in postpartum patients with SLE may protect them from hyperprolactinemia and hyperestrogenemia and may be beneficial for preventing the patients from disease relapse.

In nonpregnancy SLE patients, some studies had been performed to determine whether there is an association between hyperprolactinemia and lupus activity, but the results were controversial. Orbach et al. [17] reported that hyperprolactinemia in lupus patients was associated with serositis and anemia but not with SLE disease activity. But most other researches indicated that hyperprolactinemia was found to be associated with SLE disease activity and conventional immunosuppressive therapy decreased PRL levels as well as SLE activity [18], and elevated serum bioactive prolactin concentrations in SLE patients were associated with disease activity [19]. Serum prolactin includes several isoforms, most of them are free PRL (small prolactin, molecular weight 23 kDa) and lesser amounts are big prolactin and big big prolactin (molecular weight 45–50 and >100 kDa, resp.) [20]. Big big PRL or macroprolactin is a PRL variant with reduced bioactivity that may contribute to the lower disease activity and absence of symptoms in SLE [18, 19]. So the varied results in different studies about the relationship of prolactin and SLE disease activity may be due to the different bioassay methods that are used to access the serum level of prolactin. In the current study, what we measured was the total PRL, including free PRL, big-, and macroprolactin. Further research work should be done to address the proportion of inactive prolactin in patient serum in the future.

Bromocriptine is a dopamine receptor agonist that could inhibit prolactin secretion and reduce serum prolactin level. It could also induce humoral and cell-mediated immunosuppression, directly modulate T and B cell function through the dopamine receptor, and reduce IFN production by macrophages [21, 22]. Recently, a study showed that bromocriptine could effectively prevent maternal and fetal complications, including premature rupture of membrane, low birth weight, preterm deliveries and decrease disease activity measured by SLE Pregnancy Disease Activity Index (SLEPDAI) [23].

For postpartum lupus patients, our study showed that oral bromocriptine therapy after delivery could not only reduce serum prolactin level quickly but also be helpful to decrease serum estradiol concentration to pregestation level rapidly. In bromocriptine treatment group, the serum prolactin levels were much lower than the control group at the second week after delivery. The estradiol levels in treatment group were lower than the control group as well. The results of survival analysis in this study confirmed that two weeks of oral bromocriptine therapy could reduce the SLE activity in treatment group even in one year after delivery.

Prolactin could modulate the immune function in SLE obviously but has little influence on the immune system of normal people [24]. The adverse impacts of estradiol on SLE have been well known by rheumatologists. One study [25] indicated that hyperprolactinemia concurrence with hyperestrogenemia may have synergistic effects on SLE and estrogen could increase the production of anti-DNA autoantibody by B cell when exposed to prolactin. So, the results of our study indicated that oral bromocriptine therapy may be beneficial to pregnant SLE patients by eliminate the adverse impacts of estradiol.

An increasing body of evidences indicated that bromocriptine is a safe drug, even for the patients during pregnancy. In the current study, no severe adverse events were found in patients taking bromocriptine, and vertigo and nausea were the most common complains. Another study [23] about using bromocriptine during pregnancy in SLE patients also showed that only 2 patients had mild headache and no severe adverse event or birth defect was observed. In more than 6000 pregnancies in women taking bromocriptine for hyperprolactinemia, the incidence of congenital malformations or abortions was not increased [26]. The endocrine society clinical practice guideline for diagnosis and treatment of hyperprolactinemia [27] also recommends bromocriptine therapy in patients who experience symptomatic growth of a prolactinoma during pregnancy.

In conclusion, the current study preliminarily indicates that oral-taking bromocriptine for two weeks in postpartum SLE patients may eliminate the effects of hyperprolactinemia and hyperestrogenemia on disease activity. In addition to the minimal side effects and low cost of treatment, prescribing lupus patients with bromocriptine has potential benefits in preventing postpartum disease relapses in one year after delivery. However, multicenter clinical trials with large sample size should be performed to evaluate the benefit of clinical application of bromocriptine.

## Figures and Tables

**Table 1:** Baseline demographic features, clinical findings, and SLEDAI scores in the pregnant SLE patients.

	Treatment group (*n* = 38)	Control group (*n* = 38)	*P* value
Age (years, mean ± SD)	30.47 ± 4.33	30.02 ± 3.95	0.57
Disease duration (years, mean ± SD)	3.27 ± 0.91	3.45 ± 1.12	0.63
Inactive SLE (%)	8 (21.05%)	8 (21.05%)	1.00
C3 (g/L, mean ± SD)	0.56 ± 0.29	0.58 ± 0.21	0.51
C4 (g/L, mean ± SD)	0.13 ± 0.06	0.14 ± 0.05	0.54
SLEDAI score (mean ± SD)	7.13 ± 1.37	6.92 ± 1.98	0.43

SLEDAI: systemic lupus erythematosus disease activity index.

**Table 2:** Comparison of the serum prolactin/estradiol levels between two groups at the second week and the second month after delivery.

Serum level	Treatment group (*n* = 38)	Control group (*n* = 38)	*P*
Prolactin (*μ*g/L, mean ± SD)			
At the second week after delivery	8.6 ± 5.0	72.6 ± 32.6	<0.001
At the second month after delivery	11.5 ± 7.1	25.7 ± 37.6	<0.05
Estradiol (ng/L, mean ± SD)			
At the second week after delivery	78.3 ± 27.3	159.5 ± 132.0	<0.001
At the second month after delivery	77.7 ± 33.6	104.7 ± 80.1	<0.05

**Table 3:** Change of SLEDAI within 1 years after delivery (mean ± SD).

SLEDAI score	Treatment group (*n* = 38)	Control group (*n* = 38)	*P*
Baseline data, mean ± SD	7.13 ± 1.37	6.92 ± 1.98	0.43
At the 6th month after delivery, mean ± SD	4.25 ± 1.28	5.85 ± 1.76	<0.001
At the 12th month after delivery, mean ± SD	3.42 ± 0.95	4.53 ± 1.15	<0.05

**Table 4:** Cumulative doses of corticosteroid and immunosuppressive agents for SLE in 12 months after delivery.

Medicines	Treatment group (*n* = 38)	Control group (*n* = 38)	*P*
Prednisone (g, mean ± SD)	3.92 ± 1.81	8.78 ± 3.84	<0.001
Cyclophosphamide (g, mean ± SD)	1.57 ± 0.91	4.32 ± 2.03	<0.001
Methotrexate (mg, mean ± SD)	99.28 ± 41.25	93.72 ± 42.01	0.39
Azathioprine (g, mean ± SD)	3.42 ± 1.90	3.27 ± 1.74	0.82
